# ^177^Lu-labelled macrocyclic bisphosphonates for targeting bone metastasis in cancer treatment

**DOI:** 10.1186/s13550-016-0161-3

**Published:** 2016-01-16

**Authors:** Ralf Bergmann, Marian Meckel, Vojtěch Kubíček, Jens Pietzsch, Jörg Steinbach, Petr Hermann, Frank Rösch

**Affiliations:** Institute of Radiopharmaceutical Cancer Research, Helmholtz-Zentrum Dresden-Rossendorf, Dresden, Germany; Institute of Nuclear Chemistry, Johannes Gutenberg University of Mainz, Fritz-Strassmann-Weg 2, 55128 Mainz, Germany; Faculty of Science, Department of Inorganic Chemistry, Charles University Prague, Prague, Czech Republic

**Keywords:** Bisphosphonate, Bone metastases, ^177^Lu, DO2A, DOTA, Biodistribution, Theranostics

## Abstract

**Background:**

Metastatic bone lesion is a common syndrome of many cancer diseases in an advanced state. The major symptom is severe pain, spinal cord compression, and pathological fracture, associated with an obvious morbidity. Common treatments including systemic application of bisphosphonate drugs aim on pain reduction and on improving the quality of life of the patient. Particularly, patients with multiple metastatic lesions benefit from bone-targeting therapeutic radiopharmaceuticals. Agents utilizing beta-emitting radionuclides in routine clinical praxis are, for example, [^89^Sr]SrCl_2_ and [^153^Sm]Sm-EDTMP. No-carrier-added (n.c.a.) ^177^Lu is remarkably suitable for an application in this scope.

**Methods:**

Five 1,4,7,10-tetraazacyclododecane N,N′,N′′,N′′-tetra-acetic acid (DOTA)- and DO2A-based bisphosphonates, including monomeric and dimeric structures and one 1,4,7-triazacyclononane-1,4-diacetic acid (NO2A) derivative, were synthesized and labelled with n.c.a. ^177^Lu. Radio-TLC and high-performance liquid chromatography (HPLC) methods were successfully established for determining radiochemical yields and for quality control. Their binding to hydroxyapatite was measured in vitro. Ex vivo biodistribution experiments and dynamic in vivo single photon computed tomography (SPECT)/CT measurements were performed in healthy rats for 5 min and 1 h periods. Data on %ID/g or standard uptake value (SUV) for femur, blood, and soft-tissue organs were analyzed and compared with [^177^Lu]citrate.

**Results:**

Radiolabelling yields for [^177^Lu]Lu-DOTA and [^177^Lu]Lu-NO2A monomeric bisphosphonate complexes were >98 % within 15 min. The dimeric macrocyclic bisphosphonates showed a decelerated labelling kinetics, reaching a plateau after 30 min of 60 to 90 % radiolabelling yields. All ^177^Lu-bisphosphonate complexes showed exclusive accumulation in the skeleton. Blood clearance and renal elimination were fast. SUV data (all for 1 h p.i.) in the femur ranged from 3.34 to 5.67. The bone/blood ratios were between 3.6 and 135.6, correspondingly. ^177^Lu-bisphosphonate dimers showed a slightly higher bone accumulation (SUV_femur_ = 4.48 ± 0.38 for [^177^Lu]Lu-DO2A(P^BP^)_2_; SUV_femur_ = 5.41 ± 0.46 for [^177^Lu]Lu-DOTA(M^BP^)_2_) but a slower blood clearance (SUV_blood_ = 1.25 ± 0.09 for [^177^Lu]Lu-DO2A(P^BP^)_2_; SUV_blood_ = 1.43 ± 0.32 for [^177^Lu]Lu-DOTA(M^BP^)_2_).

**Conclusions:**

Lu-complexes of macrocyclic bisphosphonates might become options for the therapy of skeletal metastases in the near future, since they show high uptake in bone together with a very low soft-tissue accumulation.

## Background

Bone-seeking radiopharmaceuticals showed promising results in the last decades both for diagnosis and therapy [1]. The mechanism of the therapy effect is the synergy of an enhanced accumulation of osteotropic agents on the metastatic lesion and the energy deposit by particle radiation (*β*^−^, inner conversion or *α*-particles). One of the earliest therapeutic concepts was the administration of ^89^SrCl_2_ as a calcium mimetic [2]. While ^89^Sr showed unfavorable nuclear properties in terms of *β*-energy and half-life (*β*_max_ = 1.5 MeV, *t*_1/2_ = 50 days) [3], new radiopharmaceuticals, like [^153^Sm]Sm-EDTMP were utilized. More radionuclides are under consideration such as IC-transforming ^117m^Sn. Recently, *α*-emitting [^223^Ra]RaCl_2_ was introduced [1].

The potential of lanthanide radionuclides for bone targeting was recognized early [4]. Skeletal ^177^Lu-images were obtained with the Anger camera already in the 1960s [5]. The *γ*-photons of 113 keV (6.4 %) and 208 keV (11.4 %) [6] from the ^177^Lu decay are suitable for single photon computed tomography (SPECT), whereas today the focus of ^177^Lu-compounds is mainly on the therapeutic benefit of the *β*^−^-emission. ^177^Lu-labelled somatostatin analogues are successfully used in the treatment of neuroendocrine tumors in the peptide receptor radionuclide therapy (PRRT) [7]. The ideal nuclide properties of a half-life of 6.65 days and the maximum *β*-energy of 0.497 MeV [6] together with the carrier-free production route make ^177^Lu an interesting candidate in the treatment of skeletal metastases.

In the early times of radiopharmacy, it was discovered already that the addition of chelating agents alters the biological distribution of lanthanides from primary liver uptake to almost exclusively bone accumulation [5]. “Bone seeking” polycarboxy-polyphosphates like ethylenediamine tetra(methylene phosphonic acid) (EDTMP) showed promising first results [8, 9]. Due to their low kinetic stability with lanthanide ions [10], a high EDTMP concentration in the blood pool is necessary for a stable complexation in vivo, which consequently creates a high amount of ligand carrier [11]. As opposed to these open-chain chelators, macrocyclic chelators like 1,4,7,10-tetraazacyclododecane N,N′,N″,N″-tetra-acetic acid (DOTA) show a very strong thermodynamic and kinetic stability with lanthanides [12, 13]. Even equimolar metal to DOTA ratios guarantee for complexation and in vivo stability. Initial experiments with phosphonic acid derivatives (DOTP) of DOTA complexed with ^177^Lu showed sufficient bone accumulation and low uptake in soft tissue [14].

**Chart 1:**
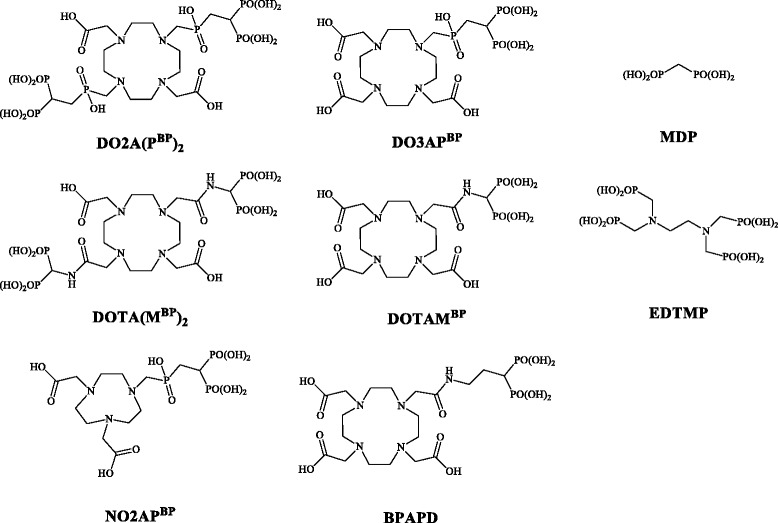
Structure of ligands discussed in this paper

An ideal chelator-based positron emission tomography (PET) nuclide is the generator-derived ^68^Ga. Phosphonate-based macrocycles for ^68^Ga complex formation such as [^68^Ga]EDTMP and [^68^Ga]DOTP showed only disappointing results in accumulating bone structures, in contrary to the ^177^Lu outcomes [15, 16]. The first compound, which yielded excellent ^68^Ga-PET, was the DOTA-based bisphosphonate BPAMD (for formula, see Chart 1) [17, 18]. It is known that bisphosphonates in general have a high affinity to calcified tissues and a very long biological half-life in the skeleton [19]. [^68^Ga]BPAMD PET/CT showed uptake values on disseminated bone metastases as high as ^18^F-fluoride, sometimes even superior [20]. Recently, the NOTA-derivative [^68^Ga]NO2AP^BP^ was identified to show even improved imaging quality [21]. In a previous work, we investigated the potential of different ^68^Ga-labelled DOTA-conjugated bisphosphonates in an animal model as PET imaging agents [22]. The logical step is the following investigation in its potential as therapeutic agents, when labelled with ^177^Lu. Nevertheless, we believe that there still are various options to chemically modify those chelate structures in order to maximize uptake on bone metastases and to further reduce soft-tissue accumulation. The current question, discussed in the present paper, is whether analogue macrocyclic bisphosphonate ligands can directly be converted from ^68^Ga complexes to ^177^Lu analogues, i.e., retaining organ uptake and pharmacology profiles known from Ga(III) structures, or whether an alternative ligand chemistry is needed to match the coordination features of Lu(III).

**Fig. 1:**
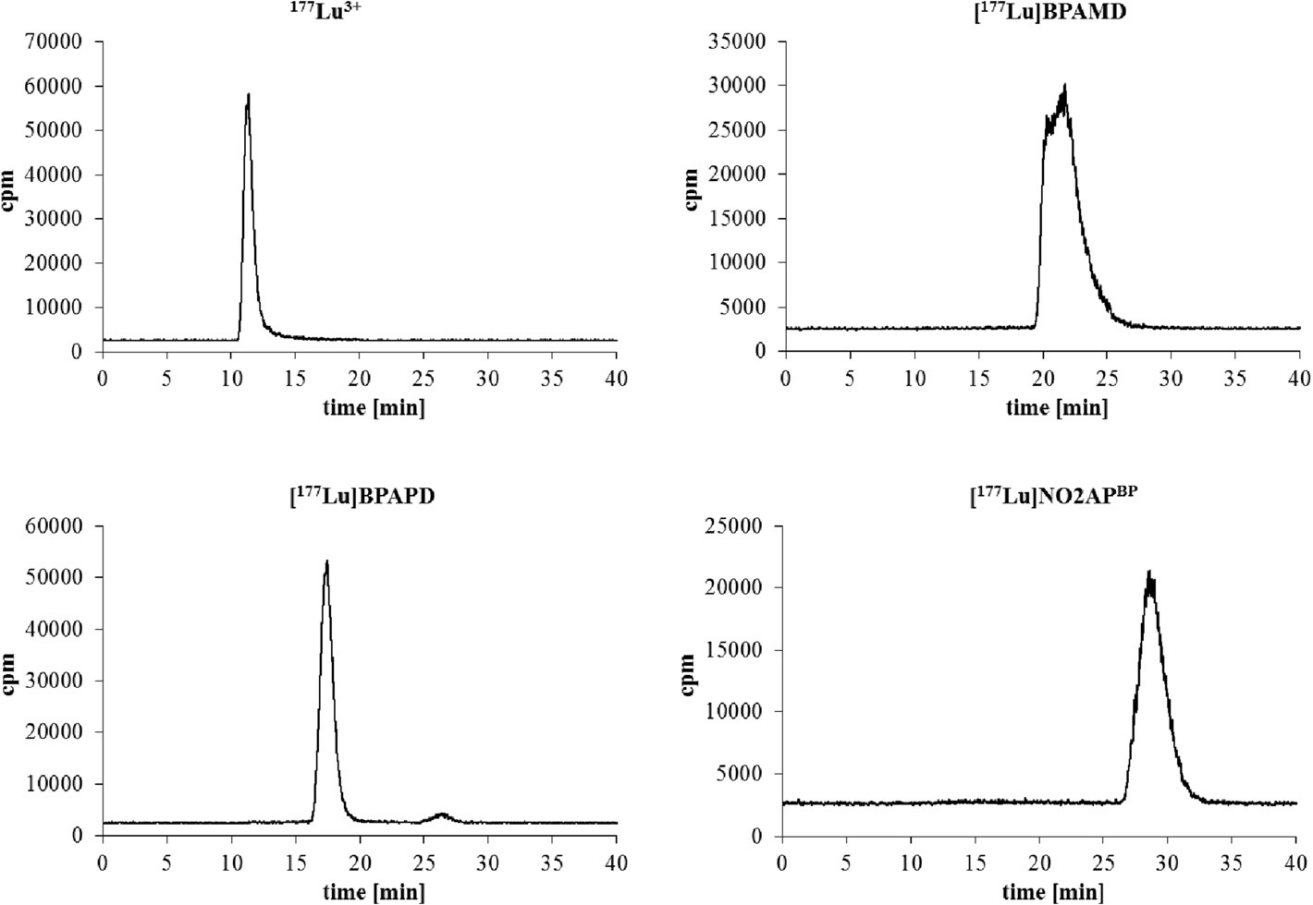
Radio-HPLC chromatograms of [^177^Lu]BPAMD, [^177^Lu]BPAPD, and [^177^Lu]NO2AP^BP^ as a representative for ^177^Lu-labelled bisphosphonates in comparison to ^177^Lu^3+^

**Fig. 2:**
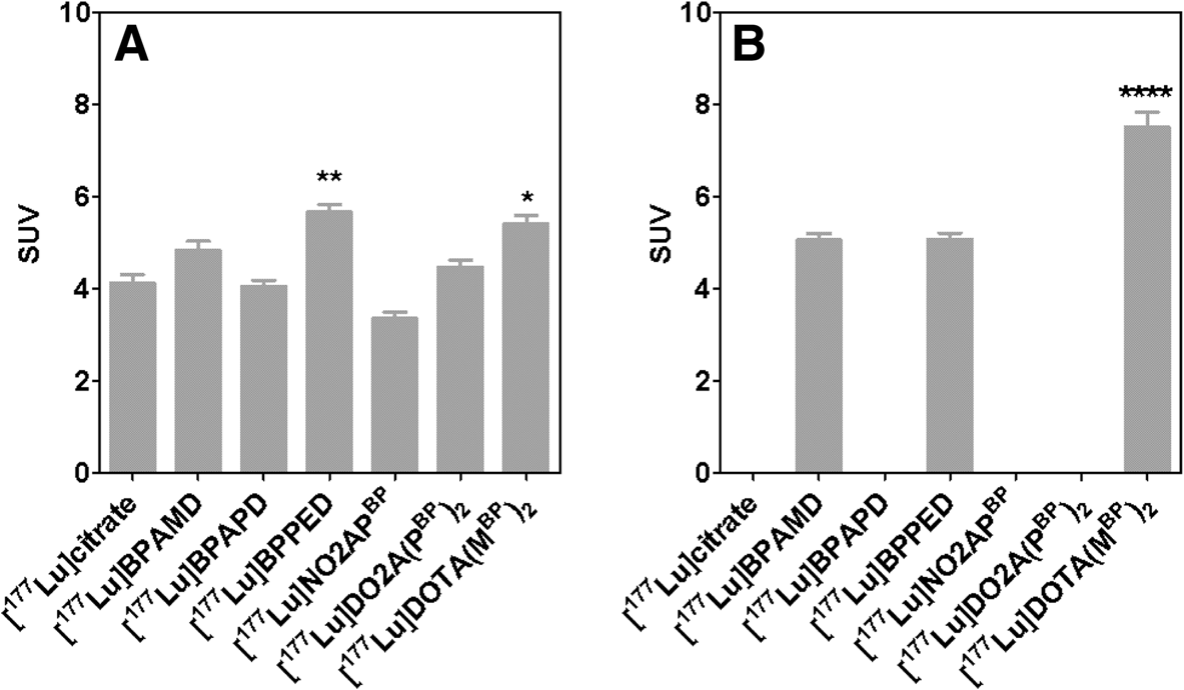
Accumulation of ^177^Lu-labelled compounds **a** 60 min and **b** 8 days p.i. in the femur. Values are mean ± SD (SUV) of the activity concentrations in both femurs of **a** four and **b** five healthy Wistar rats. In **a**, the accumulation in the femur was compared between [^177^Lu]citrate and the other radiotracers and in **b** between [^177^Lu]DOTA(M^BP^)_2_ and the other radiotracers. The *P* values are *<0.05; **<0.01, and ****<0.0001

**Fig. 3:**
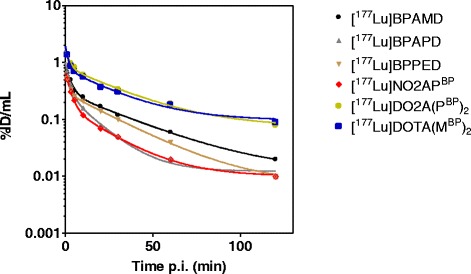
Time activity concentration curves of [^177^Lu]BPAMD, [^177^Lu]BPAPD, [^177^Lu]BPPED, [^177^Lu]NO2AP^BP^, [^177^Lu]DO2A(P^BP^)_2_, and [^177^Lu]DOTA(M^BP^)_2_ in arterial blood of Wistar rats after single intravenous injection, respectively

**Fig. 4:**
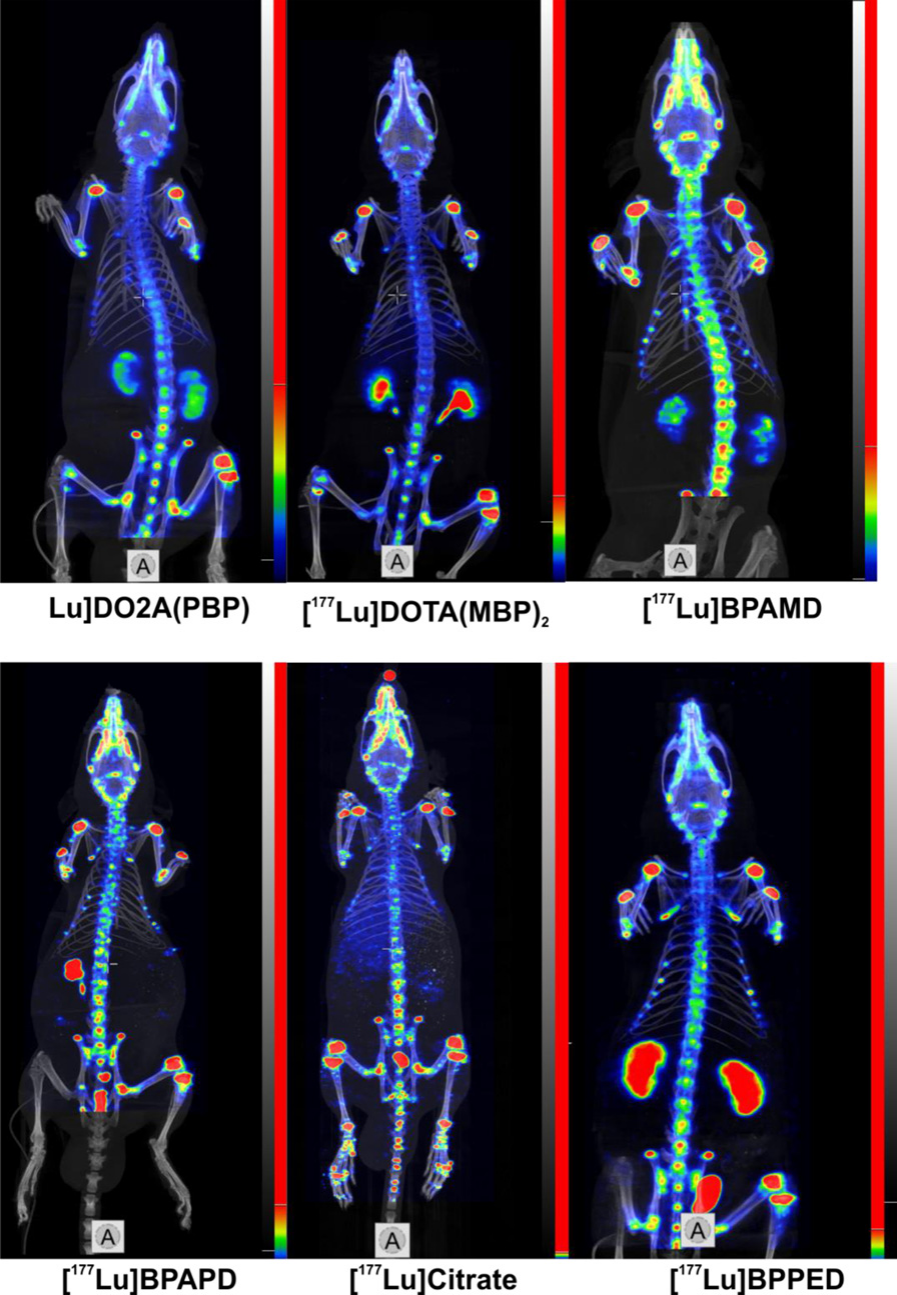
MIP of [^177^Lu]citrate, [^177^Lu]BPAMD, [^177^Lu]BPAPD, [^177^Lu]BPPED, [^177^Lu]NO2AP^BP^, [^177^Lu]DO2A(P^BP^)_2_, and [^177^Lu]DOTA(M^BP^)_2_ in Wistar rats after 1 h of single intravenous injection, respectively

**Fig. 5:**
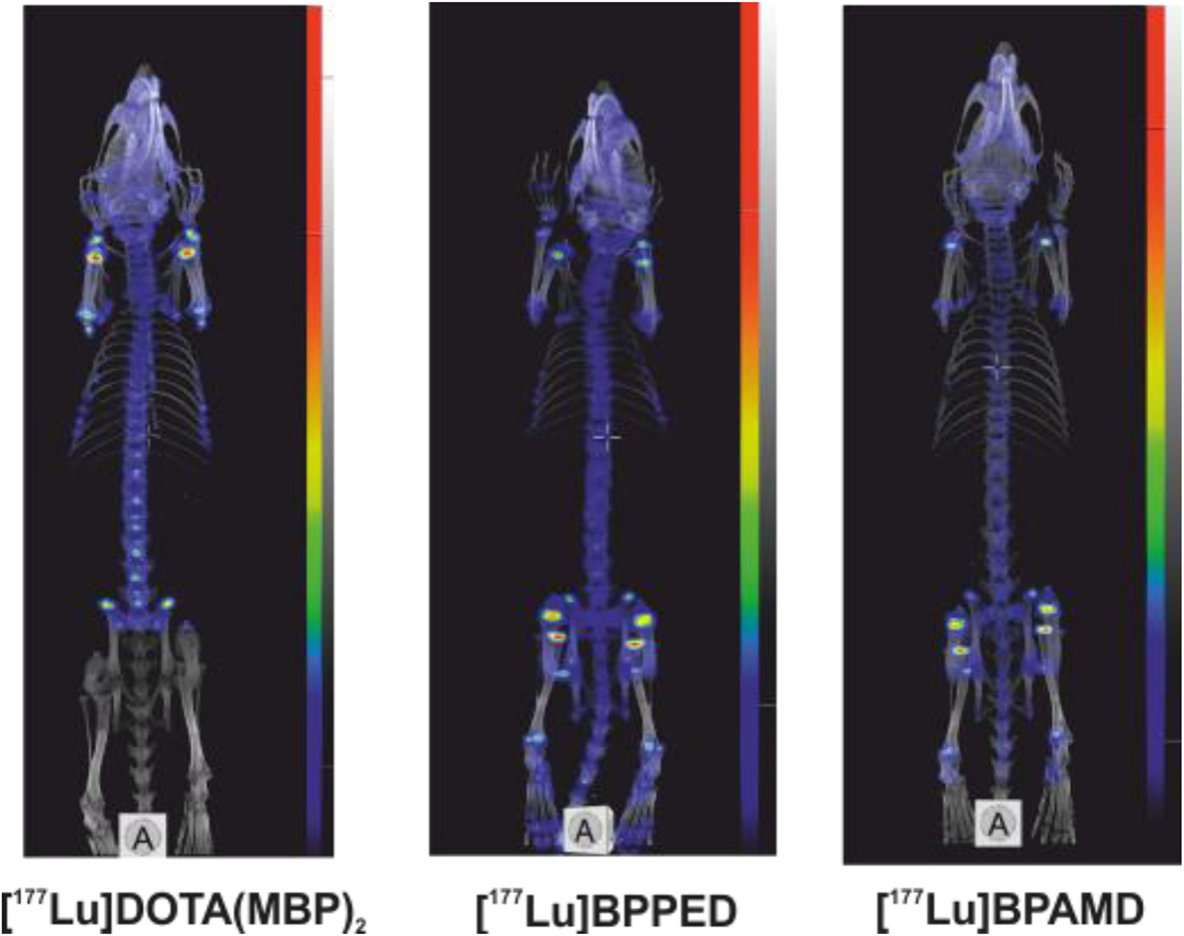
MIP of [^177^Lu]BPAMD, [^177^Lu]BPPED, and [^177^Lu]DOTA(M^BP^)_2_ in Wistar rats after 8 days of single intravenous injection, respectively

Currently, several new macrocyclic bisphosphonates are intensely discussed in the literature as candidates for complex formation with ^177^Lu and ^90^Y and subsequent “bone seeking” parameters, including hydroxy bisphosphonates [23] and phosphinates (Chart 1) [24]. In this paper, the biodistribution and bone accumulation of various macrocyclic bisphosphonates, including two dimeric DOTA and one 1,4,7-triazacyclononane-1,4-diacetic acid (NO2A) compounds (Chart 1), were studied in a healthy rat model. The ligand synthesis, in particular the synthesis of the new dimeric bisphosphonates DO2A(P^BP^)_2_ and DOTA(M^BP^)_2_ as well as labelling with ^177^Lu and radio-analytics, are reported. All the ^177^Lu species were investigated in healthy rats in in vivo small animal SPECT and ex vivo organ distribution (5 min to 1 h p.i.) studies and SUV data or %ID/g data were determined (*n* = 4) of the interested organs.

## Methods

### Chemicals

Chemicals and solvents were commercially available in analytical or high-performance liquid chromatography (HPLC) grade and were purchased from Sigma-Aldrich or Merck KGaA.

### Analytics

Proton nuclear magnetic resonance (^1^H-NMR) spectra were recorded on a Bruker 300, and ^31^P-NMR were recorded on a Bruker 600. NMR shifts were referenced to internal TMS or externally referenced to 85 % aq. H_3_PO_4_ signal. Mass spectra were recorded on an Agilent Technologies 6130 Quadrupole LC/MS spectrometer with ESI as ion source in positive or negative modes. TLC analyses of the ligand and intermediates during their synthesis were carried out with silica on aluminum foil (Merck).

Radio-TLC analyses of labelled compounds were carried out with silica on aluminum foil (Merck) or RP18 on alumina and a Canberra Packard Instant Imager. Radio-HPLC was performed on a Hewlett Packard Series 1100 with a Raytest (Radeberg, Germany) Ramona radiodetector. Radioactivity of samples was measured with an Aktivimeter Isomed 2010, MED (Nuklear-Medizintechnik Dresden GmbH).

### Radionuclides

No-carrier-added (n.c.a.) ^177^Lu was produced via the ^176^Yb-based neutron capture pathway provided by ITG (Garching, Germany) as LuCl_3_ in 0.04 M HCl [25].

### Synthesis of the dimeric bisphosphonates DOTA(M^BP^)_2_ and DO2A(P^BP^)_2_

1,4,7,10-tetraazacyclododecane-1,7-bis-tert.butyl diacetic acid ester (tert.butyl-DO2A) [26], tetraethyl chloro-acetamidomethyl-bis(phosphonate) (*1*), tetraethyl{[(ethoxyhydrophosphoryl)methyl]methylene} bis(phosphonate) (*4*) and the compounds DOTAM^BP^, DO3AP^BP^, DOTAM^EBP^, and NO2AP^BP^ were synthesized according to the published literature [21, 27, 28].

#### DOTA(M^BP^)_2_ in ester form (*2*)

Tert.butyl-DO2A (200 mg, 0.5 mmol) was dissolved in 50 mL dry acetonitrile. Potassium carbonate (10 eq., 5 mmol, 691 mg) was added, and the mixture was heated to 40 °C. Tetraethyl chloro-acetamidomethyl-bis(phosphonate) (*1*) (5 eq., 2.5 mmol, 948 mg) was dissolved in 25 mL dry acetonitrile and added dropwise to the DO2A solution. The reaction mixture was kept at 40 °C for 24 h under stirring and argon atmosphere. Activated charcoal was added, the solution was filtered, and the solvent was removed under reduced pressure. The crude product was purified by column chromatography (silica phase using a solvent mixture of NH_4_OH:MeOH:EA (1:4:15). *R*_f_ = 0.2–0.4) resulting in a yellow oil (308.5 mg, 57 %). ^1^H-NMR (CDCl_3_, 300 MHz): *δ* 1.37 (m, 24H, CH_2_–*CH*_*3*_), 1.46 (s, 18H, *t-Bu*), 2.6–3.0 (bs, 8H, cyclen– *CH*_*2*_–N), 3.1–3.5 (bs, 8H, cyclen– *CH*_*2*_–N), 3.5 (s, 8H, N–*CH*_*2*_–CO), 4.23 (m, 8H, *CH*_*2*_–CH_3_), ^31^P-NMR (CDCl_3_, 162.05 MHz): *δ* 7.78 (s, 2P). ESI-MS(+): cald 1086.49 obsd 1087.50 (M + H^+^), 1109.48 (M + Na^+^).

#### DOTA(M^BP^)_2_ (*3*)

The protected compound (*2*) (308.5 mg, 0.29 mmol) was dissolved in dry dichloromethane, and trimethylsilylbromide (20 eq., 6 mmol, 920 mg) was added at room temperature under argon atmosphere. The reaction mixture was stirred overnight, and volatiles were removed under reduced pressure. The resulting red oil was dissolved in methanol and stirred for 5 h until the solvent was removed. The residue was kept under high vacuum to remove volatiles until no change in weight was observed. A subsequent 2:1 mixture of TFA/DCM was added and the mixture was stirred overnight under argon atmosphere. After removing the solvents under reduced pressure, the crude compound was purified two times by recrystallization from boiling water. The precipitating white solid was filtered, washed with ethanol, and dried in vacuum with a yield of 140 mg (64 %). ^1^H-NMR (D_2_O/NaOD, 300 MHz): *δ* 2.84 (bs, 8H, cyclen–*CH*_*2*_–N), 2.92 (bs, 8H, cyclen–*CH*_*2*_–N), 3.37 (bs, 4H, N–*CH*_*2*_–CO), 3.64 (bs, 4H, N–*CH*_*2*_–CO), 4.38 (*t*, 2H, P–*CH*–P, ^2^*J*_PH_ = 18.22 Hz). ^31^P-NMR (H_2_O/NaOD, 162.05 MHz): *δ* 13.5. ESI-MS(–): cald 750.42 obsd 749.21 (M − H^+^), 374.10 (M − 2H^+^).

#### DO2A(P^BP^)_2_ in ester form (*5*)

To a solution of bisphosphonate (*4*) (2.4 g, 6 mmol) in 70 mL dry toluene, 601 mg tert.butyl-DO2A (0.25 eq., 1.5 mmol) and 180 mg (1 eq., 6 mmol) paraformaldehyde were added. The reaction solution was stirred under reflux for 24 h. The solvent was removed under reduced pressure, and the residue was purified by column chromatography (silica gel, NH_4_OH:MeOH:EA (1:4:15). *R*_f_ = 0.2–0.3) resulting in 625 mg (35 %) of a pale yellow oil. ^31^P-NMR (CDCl_3_, 162.05 MHz): δ 22.3 (m, 4P), 36.3 (m, 2P). ESI-MS(+): cald 1212.52 obsd 1213.51 (M + H^+^), 1236.53 (M + Na^+^).

#### DO2A(P^BP^)_2_ (*6*)

The protected bisphosphonate (*5*) (625 mg, 0.52 mmol) was dissolved in 50 mL 6 M aqueous HCl and kept at a temperature of 100 °C for 24 h. The solvent was evaporated under reduced pressure, and the excess of HCl was removed by repetitive co-evaporation from deionized water. The crude product was passed over a strong cation exchanger (Dowex 50, H^+^-form), and the aqueous solution was dried by lyophilization. The white solid was further purified by recrystallization from boiling water. Precipitation was initiated by adding small volumes of ethanol. The white solid (337 mg, 79 %) was filtered, washed with ethanol, and dried in vacuum. ^1^H-NMR (D_2_O/NaOD, 300 MHz): δ 2.56 (m, 4H –*CH*_*2*_–CH), 3.1 (bs, 8H, cyclen–*CH*_*2*_–N), 3.3 (bs, 8H, cyclen–*CH*_*2*_–N), 3.4 (bs, 4H, N–*CH*_*2*_–P) 3.5 (m, 2H, P–*CH*–P) 3.7 (bs, 4H, N–*CH*_*2*_–CO). ^31^P-NMR (D_2_O/NaOD, 162.05 MHz): *δ* 22.01 (s, 4P), 43.50 (s, 2P). ESI-MS(–): cald 820.08 obsd 819.04 (M − H^+^), 409.00 (M – 2H^+^).

#### Radiolabelling with n.c.a. ^177^Lu and quality control

Labelling of bisphosphonates with n.c.a. ^177^Lu was performed in 0.1 M sodium acetate buffer at pH = 4, by adding four volume equivalents of buffer to a solution of ^177^LuCl_3_ in 0.05 M HCl. A 10 M excess, based on ^177^Lu concentration of bisphosphonate containing chelator, was added, and the solution was kept on a thermo shaker at 98 °C up to 30 min. [^177^Lu]citrate was prepared by adding 100 μL ^177^LuCl_3_ in 0.05 M HCl to 100 μL of 0.25 M citrate buffer. Radiochemical yields (RCY) were determined by radio-HPLC (Zorbax 300SB-C18, 9.4 × 250 mm 5 μ, solvent: 100 mM tetraethylammonium phosphate pH = 2.24, 1 mL/min isocratic flow) and were cross-checked by two radio-TLC methods (silica, solvent: 0.1 M citrate buffer pH 4 and RP18 on alumina, solvent: 100 mM tetraethylammonium phosphate pH = 2.24 + 20 % ACN).

#### Animal studies

The experiments were realized corresponding to the German animal welfare regulations and institutional guidelines and with the permission of the local animal research committee at the Landesdirektion, Dresden. All experimental procedures are following the guidelines of the *European Convention for the Protection of Vertebrate Animals used for Experimental and other Scientific Purposes* (*ETS No. 123*). Male Wistar rats weighing 157.69 ± 17.10 g (mean ± SD, *N* = 35) were purchased from Unilever (HsdCpb:WU, Harlan Winkelmann, Borchen, Germany). Anesthetization was initiated and maintained by desflurane, and animals were put in the supine position and placed on a heating pad to maintain body temperature. A needle catheter was used for administration of the tracers in the tail vein. A second catheter was introduced into the right femoral artery for the extraction of blood samples for metabolite analysis, which was routinely done during SPECT/CT measurements.

#### Ex vivo biodistribution

The male Wistar rats received a short-term anesthetization by desflurane inhalation. Mean activities of 2.36 ± 0.27 MBq/kg of the ^177^Lu-labelled tracers in isotonic saline were injected in a volume of 0.5 mL in the tail vein, with the following specific details: [^177^Lu]citrate: 2.56 ± 0.07 MBq/kg, 128.58 body weight (BW, g), *n* = 4 (g); [^177^Lu]BPAMD: 2.33 ± 0.11 MBq/kg, 159.41 BW (g), *n* = 4 (g); [^177^Lu]BPAPD: 2.58 ± 0.24 MBq/kg, 156.38 BW (g), *n* = 4 (g); [^177^Lu]BPPED: 2.27 ± 0.25 MBq/kg, 153.43 BW (g), *n* = 4 (g); [^177^Lu]NO2AP^BP^: 2.52 ± 0.25 MBq/kg, 173.82 BW (g), *n* = 4 (g); [^177^Lu]DOTA(M^BP^)_2_: 1.80 ± 0.10 MBq/kg, 181.64 BW (g), *n* = 4 (g); [^177^Lu]DO2A(P^BP^)_2_: 2.46 ± 0.15 MBq/kg, 150.60 BW (g), *n* = 4 (g). The compounds [^177^Lu]DOTA(M^BP^)_2_, [^177^Lu]BPPED, and [^177^Lu]BPAMD were further chosen for a biodistribution study after 8 days p.i. A mean activity of 19.0 ± 2.5 MBq/kg was injected. Animals were sacrificed after 5 min, 60 min, and 8 days p.i. Organs of interest were excised rapidly and weighed, and the ^177^Lu activity was determined with a Wallac WIZARD automatic gamma counter (PerkinElmer, Germany) and decay corrected. The distribution data are expressed in %ID or are normalized to the BW as standard uptake value (SUV), which was calculated with the formula: SUV = (activity per g tissue)/(injected activity) × BW. The skeleton weight was calculated using the following: skeleton weight = 9.66 + 0.0355 × BW, and the total activities associated with the skeleton were calculated by using the activity concentration in the femur and the total skeleton weight [29].

#### Metabolite analysis and in vivo stability

Arterial blood plasma samples were taken during SPECT/CT examinations after 1, 3, 5, 10, 20, 30, 60, and 120 min. p.i., and the in vivo metabolism of [^177^Lu]BPAMD, [^177^Lu]BPAPD, [^177^Lu]BPPED, [^177^Lu]NO2AP^BP^, [^177^Lu]DOTA(M^BP^)_2_, and [^177^Lu]DO2A(P^BP^)_2_ was analyzed. Plasma was separated by centrifugation (3 min, 11,000×*g*) followed by precipitation of the plasma proteins with ice-cold methanol (1.5 parts per 1 part plasma) and centrifugation (3 min, 11,000×*g*). The supernatants were analyzed by radio-TLC and HPLC using the abovementioned methods for quality control. Additionally, urine samples were treated in the same way prior radio-TLC and radio-HPLC analysis.

#### SPECT/CT

Tracer accumulation in vivo was monitored with a NanoScan^®^ SPECT (Mediso Medical Imaging Systems, Budapest, Hungary) scanner. CT images were obtained with a NanoScan^®^ PET/CT (Mediso Medical Imaging, Budapest Hungary) scanner. Rats were prone and head-first positioned. The animals were anesthetized by 5 % desflurane inhalation.

#### Statistical analysis

The statistical analysis was performed by using GraphPad Prism (V5.02 for Windows, GraphPad Software, San Diego California USA, www.graphpad.com). Data are expressed as an average ± standard deviation (S.D.). Two-tailed Student’s *t* test or ANOVA was usually done to compare groups of data.

## Results

### Synthesis

**Scheme 1:**
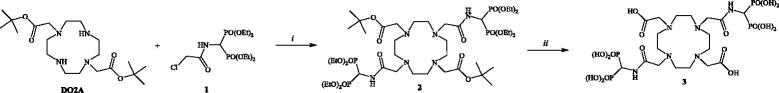
Synthesis of DOTA(M^BP^)_2_. (*i*) K_2_CO_3_, acetonitrile, 40 °C (*ii*) 1. TMS-Br, DCM, RT; 2. MeOH; 3. TFA/DCM, RT

**Scheme 2:**
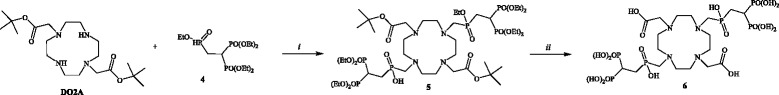
Synthesis of DO2A(P^BP^)_2._ (*i*) Toluene, H_2_CO, reflux (*ii*) 6 M HCl

The pendant arms (*1*) and (*4*) were prepared as described in literature procedures (see *experimental* part) in good yields. Alkylation of DO2A was done by a nucleophilic substitution (*2*) or by a mannich-like reaction (*5*), followed by a two-step cleavage (*3*) of the protection groups, by using trimethylsilyl bromide as a mild and efficient de-esterification agent of ethyl-protected bisphosphonates and trifluoroacetic acid for the *tert.*-butylesters of the carboxylic acid arms. Aqueous HCl (6 M) was used to de-protect the phosphinate compound (*6*) (Schemes 1 and 2). The final dimeric bisphosphonates DOTA(M^BP^)_2_ (*3*) and DO2A(P^BP^)_2_ (*6*) were obtained in moderate yields after resin and recrystallization purification.

### Radiolabelling with n.c.a. ^177^Lu and quality control

The DOTA-, DO2A-, and NO2A-conjugated bisphosphonates were successfully labelled with n.c.a. ^177^Lu(III). Yields and purities were controlled by radio-TLC and HPLC (Fig. 1) All bisphosphonates showed a very fast complexation kinetic. Since DOTA derivatives show an excellent complexation ability with lanthanides, a radiochemical yield (RCY) of more than 98 % was obtained for the abovementioned compounds within 30 min. Even the NO2A-based bisphosphonate NO2AP^BP^ showed an excellent labelling efficiency. Complexation occurred fast and quantitative (RCY > 99 %).

### Biodistribution

The ^177^Lu-labelled bisphosphonates showed a similar organ distribution with a predominant accumulation in the skeleton, cf. Tables 1, 2, and 3. Besides the bone, an appreciable amount of activity was found in the kidneys, which is reasonable according to the compound’s renal clearance. Considerable liver accumulation was only observed for [^177^Lu]citrate and [^177^Lu]DOTA(M^BP^)_2_. SUVs in the blood, liver, and kidney showed the strongest variations between the tested compounds. The highest blood levels were determined for the dimeric compound [^177^Lu]DOTA(M^BP^)_2_. The lowest blood SUV was obtained for [^177^Lu]BPAMD. Activity accumulation in the kidneys was lowest for [^177^Lu]BPAMD and [^177^Lu]BPAPD. Highest kidney concentrations were found for the dimeric bisphosphonates [^177^Lu]DO2A(P^BP^)_2_ and [^177^Lu]DOTA(M^BP^)_2_ as well as for [^177^Lu]citrate. The highest SUV determined for [^177^Lu]citrate was found in the harderian glands with a value of 9.62 ± 5.62, which is more than the doubled uptake value compared to the femur after 60 min p.i. in contrast to the tested bisphosphonates, which showed almost no uptake in the harderian glands. The data showing that the bone accumulation is completed after 60 min and is stable for 8 days. Figure 2 summerizes the bone accumulation of the investigated complexes. Figure 3 represents the activity concentration in the blood over time.

**Table 1:** Biodistribution of radioactivity after 5 min p.i. of ^177^Lu-complexes in Wistar rats

Organ	[^177^Lu]citrate		[^177^Lu]BPAMD		[^177^Lu]BPAPD		[^177^Lu]BPPED		[^177^Lu]NO2AP^BP^		[^177^Lu]DO2A(P^BP^)_2_		[^177^Lu]DOTA(M^BP^)_2_	
	Mean	SD	Mean	SD	Mean	SD	Mean	SD	Mean	SD	Mean	SD	Mean	SD
Blood	n.d.	n.d.	0.90	0.42	0.84	0.07	1.30	0.20	2.08	2.83	3.35	0.33	3.98	0.40
Brain	n.d.	n.d.	0.04	0.01	0.05	0.02	0.04	0.01	0.04	0.01	0.09	0.01	0.13	0.03
Pancreas	n.d.	n.d.	0.31	0.14	1.60	1.18	0.37	0.12	0.27	0.08	0.73	0.15	0.68	0.14
Spleen	n.d.	n.d.	0.37	0.35	1.27	0.94	0.39	0.05	0.23	0.03	0.60	0.13	0.78	0.10
Adrenal gland	n.d.	n.d.	0.40	0.13	0.55	0.17	0.70	0.41	0.60	0.42	1.23	0.19	1.07	0.19
Kidneys	n.d.	n.d.	4.53	4.01	3.61	1.81	3.09	0.48	3.08	1.14	2.87	0.29	3.10	0.54
Adipose tissue	n.d.	n.d.	0.42	0.19	1.80	0.78	0.24	0.07	0.53	0.14	1.52	0.50	0.55	0.13
Muscle	n.d.	n.d.	0.19	0.08	0.78	0.70	0.24	0.03	0.21	0.05	0.49	0.30	0.49	0.11
Heart	n.d.	n.d.	0.36	0.14	0.36	0.04	0.52	0.10	0.31	0.06	1.34	0.17	1.45	0.30
Lung	n.d.	n.d.	0.61	0.24	0.61	0.07	0.75	0.08	0.50	0.05	1.87	0.41	2.04	0.32
Thymus	n.d.	n.d.	0.29	0.11	0.30	0.06	0.38	0.08	0.22	0.04	0.76	0.09	0.93	0.14
Harderian gland	n.d.	n.d.	0.36	0.13	0.37	0.10	0.40	0.12	0.35	0.08	0.95	0.23	1.12	0.16
Liver	n.d.	n.d.	0.26	0.11	0.39	0.08	0.48	0.12	0.29	0.12	1.07	0.15	1.13	0.39
Testes	n.d.	n.d.	0.25	0.07	0.28	0.03	0.36	0.01	0.32	0.05	0.24	0.02	0.30	0.03
Femur	n.d.	n.d.	3.36	0.63	3.43	0.44	4.16	0.35	2.67	0.04	2.11	0.22	2.31	0.15

Data are expressed in SUV. Each value represents the mean (S.D.) of four animals

*n.d.* data not determined

**Table 2:** Biodistribution of radioactivity after 60 min p.i. of ^177^Lu-complexes in Wistar rats

Organ	[^177^Lu]citrate		[^177^Lu]BPAMD		[^177^Lu]BPAPD		[^177^Lu]BPPED		[^177^Lu]NO2AP^BP^		[^177^Lu]DO2A(P^BP^)_2_		[^177^Lu]DOTA(M^BP^)_2_	
	Mean	SD	Mean	SD	Mean	SD	Mean	SD	Mean	SD	Mean	SD	Mean	SD
Blood	0.22	0.11	0.04	0.01	0.04	0.00	0.06	0.01	0.07	0.02	1.25	0.09	1.43	0.32
Brain	0.06	0.01	0.01	0.00	0.01	0.00	0.01	0.01	0.01	0.01	0.04	0.01	0.05	0.01
Pancreas	0.50	0.05	0.04	0.02	0.04	0.02	0.03	0.01	0.06	0.01	0.31	0.05	0.30	0.07
Spleen	0.70	0.12	0.07	0.06	0.05	0.01	0.14	0.04	0.05	0.01	0.31	0.03	0.52	0.25
Adrenal gland	0.62	0.05	0.04	0.04	0.09	0.06	0.08	0.12	0.11	0.05	0.49	0.09	0.52	0.20
Kidneys	1.41	0.11	0.35	0.06	0.39	0.06	0.38	0.04	0.47	0.07	1.66	0.40	1.41	0.16
Adipose tissue	0.35	0.03	0.04	0.02	0.06	0.02	0.02	0.01	0.09	0.03	0.55	0.12	0.29	0.16
Muscle	0.35	0.02	0.02	0.01	0.03	0.02	0.02	0.00	0.02	0.01	0.15	0.01	0.24	0.06
Heart	0.87	0.10	0.02	0.01	0.03	0.00	0.04	0.01	0.04	0.01	0.54	0.10	0.63	0.12
Lung	1.13	0.13	0.07	0.01	0.06	0.00	0.08	0.02	0.07	0.01	0.89	0.12	0.89	0.13
Thymus	0.53	0.09	0.08	0.04	0.06	0.02	0.03	0.01	0.06	0.01	0.39	0.05	0.37	0.10
Harderian gland	9.62	5.62	0.04	0.01	0.11	0.11	0.16	0.08	0.06	0.01	0.42	0.05	0.46	0.09
Liver	1.04	0.09	0.04	0.01	0.04	0.01	0.13	0.01	0.04	0.00	0.52	0.11	0.69	0.36
Testes	0.42	0.03	0.03	0.01	0.02	0.01	0.03	0.00	0.03	0.01	0.28	0.03	0.33	0.04
Femur	4.12	0.31	4.84	0.44	4.05	0.36	5.67	0.10	3.34	0.28	4.48	0.38	5.41	0.46

Data are expressed in SUV. Each value represents the mean (S.D.) of four animals

**Table 3:** Biodistribution of radioactivity after 8 days p.i. of ^177^Lu-complexes in Wistar rats

Organ	[^177^Lu]citrate		[^177^Lu]BPAMD		[^177^Lu]BPAPD		[^177^Lu]BPPED		[^177^Lu]NO2AP^BP^		[^177^Lu]DO2A(P^BP^)_2_		[^177^Lu]DOTA(M^BP^)_2_	
	Mean	SD	Mean	SD	Mean	SD	Mean	SD	Mean	SD	Mean	SD	Mean	SD
Blood	n.d.	n.d.	0.001	0.000	n.d.	n.d.	0.001	0.001	n.d.	n.d.	n.d.	n.d.	0.003	0.001
Brain	n.d.	n.d.	0.004	0.001	n.d.	n.d.	0.014	0.021	n.d.	n.d.	n.d.	n.d.	0.018	0.007
Pancreas	n.d.	n.d.	0.006	0.001	n.d.	n.d.	0.005	0.003	n.d.	n.d.	n.d.	n.d.	0.067	0.019
Spleen	n.d.	n.d.	0.020	0.002	n.d.	n.d.	0.021	0.003	n.d.	n.d.	n.d.	n.d.	0.697	0.348
Adrenal gland	n.d.	n.d.	0.009	0.006	n.d.	n.d.	0.033	0.011	n.d.	n.d.	n.d.	n.d.	0.166	0.063
Kidneys	n.d.	n.d.	0.076	0.015	n.d.	n.d.	0.088	0.010	n.d.	n.d.	n.d.	n.d.	0.369	0.075
Adipose tissue	n.d.	n.d.	0.004	0.003	n.d.	n.d.	0.004	0.004	n.d.	n.d.	n.d.	n.d.	0.026	0.012
Muscle	n.d.	n.d.	0.136	0.107	n.d.	n.d.	0.027	0.056	n.d.	n.d.	n.d.	n.d.	0.024	0.003
Heart	n.d.	n.d.	0.006	0.001	n.d.	n.d.	0.008	0.001	n.d.	n.d.	n.d.	n.d.	0.099	0.024
Lung	n.d.	n.d.	0.008	0.001	n.d.	n.d.	0.008	0.002	n.d.	n.d.	n.d.	n.d.	0.117	0.045
Thymus	n.d.	n.d.	0.006	0.001	n.d.	n.d.	0.006	0.002	n.d.	n.d.	n.d.	n.d.	0.109	0.033
Harderian gland	n.d.	n.d.	0.105	0.127	n.d.	n.d.	0.113	0.132	n.d.	n.d.	n.d.	n.d.	0.331	0.131
Liver	n.d.	n.d.	0.025	0.002	n.d.	n.d.	0.027	0.003	n.d.	n.d.	n.d.	n.d.	0.470	0.078
Testes	n.d.	n.d.	0.004	0.000	n.d.	n.d.	0.004	0.001	n.d.	n.d.	n.d.	n.d.	0.050	0.005
Femur	n.d.	n.d.	5.039	0.453	n.d.	n.d.	5.197	0.435	n.d.	n.d.	n.d.	n.d.	7.340	1.009

Data are expressed in SUV. Each value represents the mean (S.D.) for five animals. As an exception are the data in three decimal places to show also the very low values

*n.d.* data not determined

The “bone to soft tissue” ratios are presented in Tables 4, 5, and 6. The best bone-to-muscle ratio was observed for [^177^Lu]BPPED (355.8). [^177^Lu]BPPED showed as well the best bone-to-kidney ratio. The best bone-to-liver ratio was determined for [^177^Lu]BPAMD (127.5), while the lowest ratio was obtained for [^177^Lu]citrate (4.0). The total skeleton retention was calculated as the following: starting with the lowest value of 30.5 ± 1.1 %ID for [^177^Lu]NO2AP^BP^, 39.4 ± 2.5 %ID for [^177^Lu]BPAPD, 44.6 ± 3.2 %ID for [^177^Lu]DO2A(P^BP^)_2_, 45.7 ± 6.2 %ID for [^177^Lu]citrate, 46.7 ± 5.3 %ID for [^177^Lu]BPAMD, 48.2 ± 4.1 %ID for [^177^Lu]DOTA(M^BP^)_2_, and 56.3 ± 6.7 %ID for [^177^Lu]BPPED after 60 min. The total skeleton accumulation was constant over 8 days for [^177^Lu]BPAMD and [^177^Lu]BPPED. Only the total skeletal retention of [^177^Lu]DOTA(M^BP^)_2_ increased to 64.5 ± 1.5 %ID but showed at the same time the minimal target to soft-tissue ratio.

**Table 4:** Ratios between bone and soft tissue after 5 min p.i. of ^177^Lu-complexes in Wistar rats

Bone/organ	[^177^Lu]citrate	[^177^Lu]BPAMD	[^177^Lu]BPAPD	[^177^Lu]BPPED	[^177^Lu]NO2AP^BP^	[^177^Lu]DO2A(P^BP^)_2_	[^177^Lu]DOTA(M^BP^)_2_
Blood	n.d.	3.7	4.1	3.2	1.3	0.6	0.6
Brain	n.d.	83.9	68.5	104.0	66.8	23.4	17.7
Pancreas	n.d.	10.8	2.1	11.2	9.9	2.9	3.4
Spleen	n.d.	9.1	2.7	10.7	11.6	3.5	3.0
Adrenal gland	n.d.	8.4	6.2	5.9	4.5	1.7	2.2
Kidneys	n.d.	0.7	0.9	1.3	0.9	0.7	0.7
Adipose tissue	n.d.	8.0	1.9	17.3	5.0	1.4	4.2
Muscle	n.d.	17.7	4.4	17.3	12.7	4.3	4.7
Heart	n.d.	9.3	9.5	8.0	8.6	1.6	1.6
Lung	n.d.	5.5	5.6	5.5	5.3	1.1	1.1
Thymus	n.d.	11.6	11.4	10.9	12.1	2.8	2.5
Harderian gland	n.d.	9.3	9.3	10.4	7.6	2.2	2.1
Liver	n.d.	12.9	8.8	8.7	9.2	2.0	2.0
Testes	n.d.	13.4	12.2	11.6	8.3	8.8	7.7

*n.d.* data not determined

**Table 5:** Ratios between bone and soft tissue after 60 min p.i. of ^177^Lu-complexes in Wistar rats

Bone/organ	[^177^Lu]citrate	[^177^Lu]BPAMD	[^177^Lu]BPAPD	[^177^Lu]BPPED	[^177^Lu]NO2AP^BP^	[^177^Lu]DO2A(P^BP^)_2_	[^177^Lu]DOTA(M^BP^)_2_
Blood	18.7	135.6	95.1	92.9	45.8	3.6	3.8
Brain	65.4	613.4	513.9	419.9	254.3	111.5	104.0
Pancreas	8.2	136.4	104.9	181.6	56.7	14.5	18.0
Spleen	5.8	68.2	85.7	41.0	66.0	14.2	10.4
Adrenal gland	6.6	109.6	45.8	72.5	31.4	9.1	10.4
Kidneys	2.9	14.0	10.3	14.9	7.1	2.7	3.8
Adipose tissue	11.8	134.5	70.7	269.7	37.2	8.2	18.4
Muscle	11.8	251.7	158.7	355.8	141.4	29.8	22.9
Heart	4.7	210.1	158.7	141.1	89.0	8.2	8.6
Lung	3.7	69.1	62.6	67.5	46.8	5.1	6.1
Thymus	7.7	58.6	70.7	212.0	59.0	11.6	14.4
Harderian gland	0.4	118.8	35.8	36.4	85.6	10.6	11.7
Liver	4.0	127.5	105.4	42.3	87.0	8.6	7.8
Testes	9.9	179.1	199.7	185.1	113.5	15.8	16.2

**Table 6:** Ratios between bone and soft tissue after 8 days p.i. of ^177^Lu-complexes in Wistar rats

Bone/organ	[^177^Lu]citrate	[^177^Lu]BPAMD	[^177^Lu]BPAPD	[^177^Lu]BPPED	[^177^Lu]NO2AP^BP^	[^177^Lu]DO2A(P^BP^)_2_	[^177^Lu]DOTA(M^BP^)_2_
Blood	n.d.	6703	n.d.	5123	n.d.	n.d.	2639
Brain	n.d.	1405	n.d.	365	n.d.	n.d.	419
Pancreas	n.d.	851	n.d.	1104	n.d.	n.d.	110
Spleen	n.d.	257	n.d.	246	n.d.	n.d.	11
Adrenal gland	n.d.	547	n.d.	159	n.d.	n.d.	44
Kidneys	n.d.	66	n.d.	59	n.d.	n.d.	20
Adipose tissue	n.d.	1158	n.d.	1231	n.d.	n.d.	287
Muscle	n.d.	37	n.d.	192	n.d.	n.d.	312
Heart	n.d.	885	n.d.	653	n.d.	n.d.	74
Lung	n.d.	634	n.d.	637	n.d.	n.d.	63
Thymus	n.d.	787	n.d.	933	n.d.	n.d.	67
Harderian gland	n.d.	48	n.d.	46	n.d.	n.d.	22
Liver	n.d.	203	n.d.	189	n.d.	n.d.	16
Testes	n.d.	1136	n.d.	1214	n.d.	n.d.	148

*n.d.* data not determined

### Metabolite analysis and in vivo stability

Radio-HPLC and TLC analysis of blood samples showed no evidence of any metabolization of the compounds [^177^Lu]NO2AP^BP^, [^177^Lu]BPAMD, [^177^Lu]BPAPD, and [^177^Lu]BPPED within a period of 120 min. The abovementioned compounds were found to be intact in the urine and the serum probes, and no traces of unknown ^177^Lu species or compounds were observed. The blood half-lives of the slow components (*t*_1/2_(*β*)) were 23.3 min for [^177^Lu]BPAMD, 10.4 min for [^177^Lu]BPAPD, 18.7 min for [^177^Lu]BPPED, 14.7 min for [^177^Lu]NO2AP^BP^, 20.6 min for [^177^Lu]DO2A(P^BP^)_2_, and 17.7 min for [^177^Lu]DOTA(M^BP^)_2_.

### SPECT/CT molecular imaging in vivo

Images obtained from SPECT showed that the therapeutic ^177^Lu-labelled bisphosphonates exclusively accumulated in the skeleton with a high target-to-background ratio, cf. Figs. 4 and 5. No considerable fractions of ^177^Lu activity were found in other organs after 60 min, which is consistent to the data gathered from the ex vivo biodistribution. An obvious amount of activity was present in the growth plate as well as in other articulations.

## Discussion

The DOTA-based monomeric bisphosphonates BPAMD, BPAPD, and BPPED have been successfully radiolabeled with the therapeutic *β*^−^-emitter ^177^Lu(III) in excellent yields over 98 % as well as the NO2A-based bisphosphonate NO2AP^BP^ and the dimeric compounds DOTA(M^BP^)_2_ and DO2A(P^BP^)_2_. All tracers tested showed a distinguished high accumulation on the bone surface, eminently in the epiphyseal plate. We suppose that this particular uptake in the epiphyseal areas may serve as an analogy to the tracer’s behavior on bone metastases. High uptakes were observed for the dimeric compound [^177^Lu]DOTA(M^BP^)_2_ (SUV_femur_ = 5.41 ± 0.41). All bisphosphonates underwent a renal body clearance, and the blood elimination was very fast especially for the monomeric compounds. No brain or notable liver uptake was observed. The compounds were found to be complete intact in the urine and blood.

The characteristic accumulation specific in the growth plate of the skeleton shows that the compound uptake profile is subject to the bone turnover. For that reason, the described compounds in this manuscript should be well suited for a targeted ^177^Lu therapy to osteoblastic bone metastases, where they should preferably accumulate. An important factor for a therapeutic application is the target-to-background ratio (TBR) and a fast excretion of non-target bond activity. A good TBR and a fast blood and body clearance reduces the radiation dose of the non-targeted tissue and thus will reduce toxic side effects and enhances the therapeutic efficiency and tolerance.

Although the dimeric compounds [^177^Lu]DOTA(M^BP^)_2_ and [^177^Lu]DO2A(P^BP^)_2_ showed the highest bone accumulation, their TBR was lowest, besides [^177^Lu]citrate. The additional bisphosphonate moiety may have a stronger bone-binding effect, albeit the blood levels of these compounds were significantly higher after 60 min (SUV_blood_{[^177^Lu]DOTA(M^BP^)_2_} = 1.43 ± 0.32; SUV_blood_{[^177^Lu]DO2A(P^BP^)_2_} = 1.25 ± 0.09) compared to the monomeric bisphosphonates (SUV_blood_{[^177^Lu]BPAMD} = 0.04 ± 0.01; SUV_blood_{[^177^Lu]NO2AP^BP^} = 0.07 ± 0.02). It might be the case that these higher blood concentrations are the reason for the enhanced skeletal accumulation. Because of the fast blood and renal clearance of the monomeric bisphosphonates, the time scale for target accumulation is shortened compared to the dimeric compounds. The reason for the decreased clearance of the dimers is yet not clear and it might be an effect of the higher negative charge of these compounds or may be influenced by serum protein binding. The data from ex vivo organ distribution of the dimeric bisphosphonates are also not matching with [^177^Lu]citrate. Liver uptake, blood values, and the accumulation in the harderian glands as well as in the femur are significantly different. If a therapeutic approach of bone metastases benefits from the higher skeleton accumulation of the dimeric bisphosphonates, it has yet to be evaluated in future in a dosimetry study, considering the lower TBR and the higher blood levels.

The lowest skeleton uptake was observed for the NO2A phosphinate-linked bisphosphonate [^177^Lu]NO2AP^BP^ (SUV_femur_{[^177^Lu]NO2AP^BP^} = 3.34 ± 0.28, 60 min p.i.). Contrary to the results with ^177^Lu(III), it was reported previously that [^68^Ga]NO2AP^BP^ showed an excellent bone binding with a brilliant TBR [22], remarkably suitable as a PET imaging agent. However, it is known that lanthanides require seven-dentate chelators like DOTA derivates, and the NO2A phosphinate offers only six. Since the bisphosphonate moiety is able to complex divalent metal ions like Ca^2+^, it might be the case that some parts of the phosphonate groups function as additional donors to the ^177^Lu-NO2AP complex. A partial loss of the functional bisphosphonate moiety due to stabilizing the ^177^Lu(III) complex might reduce the binding potential of the bisphosphonate group to the bone and thus reduces the affinity of the [^177^Lu]NO2AP^BP^ compound to the target tissue in contrast to the ^68^Ga(III) complex.

Highest bone accumulation of tested tracers was observed for [^177^Lu]BPPED with a SUV of 5.67 ± 0.10 in the femur after 60 min and [^177^Lu]DOTA(M^BP^)_2_ after 8 days. [^177^Lu]BPPED showed a TBR of the muscle and blood of 355.8 and 92.3, respectively. Excellent bone accumulation (SUV = 4.84 ± 0.44) as well as a superior TBRs were observed also for [^177^Lu]BPAMD, with TBR values of 127.5 to the liver and 135.6 to the blood, which was the highest for all tested compounds. [^177^Lu]BPAMD revealed a very fast blood clearance and renal excretion.

## Conclusions

Within this study, BPAMD showed to be a potential bone-targeting agent to treat skeletal metastases with ^177^Lu. Contrary to other actual discussed tracers like EDTMP, BPAMD proved to be an efficient ^68^Ga-PET imaging agent for bone metastasis [21]. Neither [^177^Lu/^153^Sm]Lu/Sm-EDTMP nor [^223^Ra]RaCl_2_ offer this *theranostic* approach. Patients may benefit from a specific [^177^Lu]BPAMD dose application, calculated from the patients’ individual uptake profile previously (pre-therapeutically) determined by [^68^Ga]BPAMD PET examinations. The same way, post-therapeutic quantitative PET studies are possible.
